# Inflammatory responses to induced infectious endometritis in mares resistant or susceptible to persistent endometritis

**DOI:** 10.1186/1746-6148-8-41

**Published:** 2012-03-29

**Authors:** Mette Christoffersen, Elizabeth Woodward, Anders M Bojesen, Stine Jacobsen, Morten R Petersen, Mats HT Troedsson, Henrik Lehn-Jensen

**Affiliations:** 1 Department of Large Animal Sciences, Faculty of Health and Medical Sciences, University of Copenhagen, Dyrlaegevej 68, Frederiksberg, Copenhagen DK-1870, Denmark; 2 The Maxwell H. Gluck Equine Research Center, University of Kentucky, Lexington, KY, USA; 3 Department of Veterinary Disease Biology, Faculty of Health and Medical Sciences, University of Copenhagen, Stigboejlen 4, Frederiksberg, Copenhagen DK-1870, Denmark; 4 Department of Large Animal Sciences, Faculty of Health and Medical Sciences, University of Copenhagen, Hoejbakkegaards Alle 5, Taastrup DK-2630, Denmark

**Keywords:** Infectious endometritis, Susceptibility, Cytokines, Serum amyloid A

## Abstract

**Background:**

The objective of the study was to evaluate the gene expression of inflammatory cytokines (interleukin [*IL*]-*1β*, *IL-6*, *IL-8*, *IL-10*, tumor necrosis factor [*TNF*]-*α*, *IL-1 *receptor antagonist [ra] and serum amyloid A (*SAA*) in endometrial tissue and circulating leukocytes in response to uterine inoculation of 10^5 ^colony forming units (CFU) *Escherichia coli *in mares. Before inoculation, mares were classified as resistant or susceptible to persistent endometritis based on their uterine inflammatory response to infusion of 10^9 ^killed spermatozoa and histological assessment of the endometrial quality. Endometrial biopsies were obtained 3, 12, 24 and 72 hours (h) after bacterial inoculation and blood samples were obtained during the 7 day period post bacterial inoculation. Expression levels of cytokines and *SAA *were determined by quantitative real-time reverse transcriptase PCR (qRT-PCR).

**Results:**

Compared to levels in a control biopsy (obtained in the subsequent estrous), resistant mares showed an up-regulation of *IL-1β*, *IL-6*, *IL-8 *and *TNF-α *at 3 h after *E. coli *inoculation, while susceptible mares showed increased gene expression of *IL-6 *and *IL-1ra*. Susceptible mares had a significant lower gene expression of *TNF-α*,*IL-6 *and increased expression of *IL-1ra *3 h after *E. coli *inoculation compared to resistant mares. Susceptible mares showed a sustained and prolonged inflammatory response with increased gene expression levels of *IL-1β*, *IL-8*, *IL-1ra and IL-1β:IL-1ra *ratio throughout the entire study period (72 h), whereas levels in resistant mares returned to estrous control levels by 12 hours. Endometrial mRNA transcripts of *IL-1β *and *IL-1ra *were significantly higher in mares with heavy uterine bacterial growth compared to mares with no/mild growth.

All blood parameters were unaffected by intrauterine *E. coli *infusion, except for a lower gene expression of *IL-10 *at 168 h and an increased expression of *IL-1ra *at 48 h observed in susceptible mares compared to resistant mares.

**Conclusions:**

The current investigation suggests that endometrial mRNA transcripts of pro-inflammatory cytokines in response to endometritis are finely regulated in resistant mares, with initial high expression levels followed by normalization within a short period of time. Susceptible mares had a prolonged expression of pro-inflammatory cytokines, supporting the hypothesis that an unbalanced endometrial gene expression of inflammatory cytokines might play an important role in the pathogenesis of persistent endometritis.

## Background

For decades, infectious endometritis has been a major cause of infertility in mares [1,2]. An inflammatory response secondary to uterine infection appear to be a major contributor to a suboptimal uterine environment [1,3] and may play a role in the pathogenesis of early embryonic loss. The ability of mares to eliminate uterine infections has been studied intensively for the past 40 years. Intrauterine infusion of bacteria (*Streptococcus equi subsp. zooepidemicus*) has been the traditional method for studying the pathogenesis of susceptibility to persistent infection and uterine defense mechanisms in mares. More than forty years ago, Hughes and Loy [4] and Peterson et al. [5] observed that mares differ in their susceptibility to uterine infections. A transient breeding-induced endometritis is a normal event immediately following breeding, and the inflammatory response is necessary for the effective removal of debris, bacteria and excess spermatozoa from the uterine lumen [6]. Several studies have shown that introduction of semen to the uterine lumen will give rise to a transient inflammatory response indistinguishable from inflammation triggered by bacteria [6-8]. In both scenarios the inflammation will activate innate- [9-13] and humoral [14,15] immune responses in the mare, which together with the physical clearance of the uterine contents serve to reestablish homeostasis [16,17]. A compromised uterine clearance caused by impaired myometrial activity leading to fluid accumulation and a sustained inflammatory response seem to be the key factors in the pathogenesis of persistent endometritis [18]. A resistant mare is capable of clearing infectious endometritis within 48 hours whereas a susceptible mare will remain infected [17]. The inflamed and infected uterine environment is incompatible with survival of the embryo at the time it descends into the uterine lumen at day 6 [19].

Cytokines play an important role in a wide range of reproductive related processes. The complexity of their network regulation is due to unique properties of cytokines, including pleitropism, where each cytokine has multiple target cells in an array of different organs and where responses may differ according to cell type [20]. An upregulated endometrial gene expression of pro- and anti-inflammatory cytokines and SAA and a systemic acute phase response (APR) have been described in mares with experimentally induced *E. coli *endometritis [9], as has gene expression of cytokines in response to artificial insemination with dead spermatozoa in resistant and susceptible mares [11,12]. Although the endometrial and systemic innate immune response has been investigated in mares with endometritis, there are no reports relating the cytokine and APR to induced infectious endometritis in mares classified as resistant or susceptible to persistent endometritis.

The aim of the present study was to evaluate a set of inflammatory factors and clinical parameters as markers of endometrial inflammation subsequent to induction of infectious endometritis in mares classified as resistant or susceptible to persistent endometritis.

## Methods

### Experimental animals

Five resistant (mean age 6 years) and 7 susceptible (mean age 19 years) mares were selected through a meticulous screening procedure of research mares of mixed breeds and used in this study. All mares were maintained at the Department of Veterinary Science's Maine Chance Farm, University of Kentucky, Lexington, KY, USA. All experimental procedures were approved by the Institutional Animal Care and Use Committee of the University of Kentucky.

### Selection of mares

**Figure 1:**
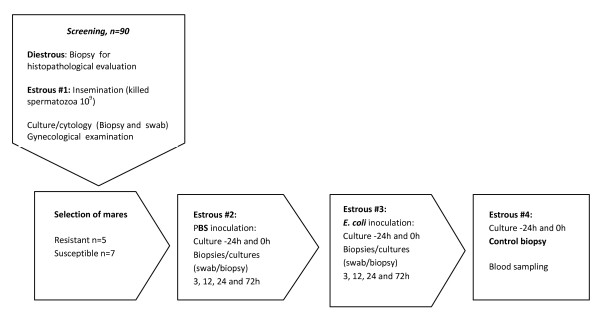
**Flow chart**. Flow chart depicting the steps and tests used for selecting resistant and susceptible mares for the study, and the timeline for PBS and *E. coli *inoculation.

A flow chart depicting the steps and tests used for selecting mares for the study, and the timeline for PBS and *E. coli *inoculation is shown in Figure 1. In total, 90 non-pregnant mares were screened for resistance and susceptibility to persistent endometritis. Out of these, 12 mares were assigned to either of two groups, resistant or susceptible, based on endometrial histology, bacteriology, cytology and response to insemination with freeze-killed stallion spermatozoa.

Uterine swab samples and endometrial biopsies were collected using an alligator jaw biopsy punch introduced into the uterus through a sterile speculum (Equivet ^®^, Kruuse A/S, Langeskov, Denmark) 3, 12, 24 and 72 h after inoculation. Biopsies were collected from the ventral part of the uterine body. The samples were immediately transported to the laboratory for preparation and analysis.

An endometrial biopsy was obtained in diestrous (day 5 post ovulation) from all mares, fixed in 10% formalin, sectioned at 5 μm and stained with hematoxylin and eosin. Each biopsy was examined for periglandular fibrosis, inflammatory cells, glandular distribution and lymphatic lacunae, then graded according to Kenney and Doig [21]. The semen was prepared from a fresh ejaculate, washed in phosphate buffered saline (PBS) (pH 7.4), resuspended in milk based semen extender (EquiPro^®^; Minitube of America, Verona, WI, USA) to yield 1 × 10^9 ^spermatozoa in 35 mL of extender per insemination dose, then stored at -20°C. Before use, the semen was thawed at room temperature. This method has previously been shown to reliably induce a uterine inflammation [22]. Once in estrus (determined by the presence of uterine edema and a follicle of at least 35 mm in size), mares were inseminated with the killed semen, and then evaluated daily. A uterine swab sample and low volume uterine lavage were obtained 48 or 96 h later depending on uterine biopsy score [21]. 250 ml of Lactated Ringer were infused into the uterus, and a minimum of 50 ml were recovered. To evaluate the mares' ability to clear uterine inflammation, uterine samples were collected at different time points based on mares' endometrial quality. In mares with a uterine biopsy I and IIa uterine samples were obtained at 48 h to confirm resistance to persistent endometritis, and at 96 h in mares with a uterine biopsy IIb or III to confirm susceptibility.

Gonadotropin releasing hormone (GnRH; Biorelease 1.5 mg, Deslorelin, BET labs, Lexington, KY, USA) was administered to induce ovulation when a 35 mm follicle and uterine edema was present.

Group I consisted of five mares found to be resistant to persistent endometritis. They had endometrial tissue histologically rated as I or IIa [21], and negative cytology (< 2 polymorphonuclear neutrophils (PMNs) per 5 fields at 400 × magnification) from a low volume lavage and negative bacterial growth obtained from a uterine swab (Minitube) and no intrauterine fluid 48 h after insemination with killed semen. Group II comprised seven mares susceptible to persistent endometritis with a grade IIb or III endometrium [21] and an impaired ability to clear uterine inflammation (> 2 PMNs per 5 fields at 400 × magnification) and intrauterine fluid 96 h after insemination with killed semen. More than 2 cm of intrauterine fluid was recorded as fluid retention.

### PBS/*E. coli *inoculation and collection of uterine samples

All mares received an intrauterine infusion of PBS in an estrous cycle prior to the *E. coli *infusion and all experimental procedures and sampling planned for the subsequent *E. coli *infusion were carried out in this estrous cycle to document the effects of repeated sampling in each individual mare. Ten ml of PBS (pH 7.4) was infused into the uterus via a sterile insemination catheter (Butler Schein Animal Health, Dublin, OH, USA) of each mare.

Mares were examined daily for follicular development, intrauterine fluid, development of uterine edema and cervical and uterine tone. In the absence of a distinct corpus luteum, the presence of a dominant follicle (> 25 mm), uterine edema and decreased uterine and cervical tone, a uterine swab sample was collected for bacterial culture, and *E. coli *was infused in presence of a > 30 mm follicle (24-48 h later). Mares were prepared as for artificial insemination immediately prior to inoculation, and a uterine swab sample was collected (0 h) for bacterial culture and cytology to verify a sterile and non-inflamed uterine environment at the time of inoculation. A total of 10^5 ^CFU of *E. coli *in 10 ml of PBS (pH 7.4) were inoculated via a sterile insemination catheter (Butler Schein Animal Health). The *E. coli *strain (241) was originally isolated from a mare with infectious endometritis, and previously used in an experimental infectious endometritis model [9].

Transrectal ultrasonography of the reproductive organs was performed -24, 0, 3, 12, 24, 48 and 72 h after inoculation with PBS/*E. coli *for detection of intrauterine fluid and ovary status. Uterine swab samples and endometrial biopsies were collected 3, 12, 24 and 72 h after inoculation. Biopsies were collected from the ventral part of the uterine body. The samples were immediately transported to the laboratory for preparation and analysis. The mares received 2500 IU of human chorionic gonadotropin (Chorulon; Intervet, Millsboro, DE, USA) as an ovulation-inducing agent when a follicle > 35 mm and pronounced uterine edema were present.

### Control biopsy

In the estrous following *E. coli *infusion, a uterine swab sample was collected from the mares to determine the presence of bacterial growth and inflammation (positive exfoliative cytology). If a sterile and non-inflamed uterine environment was detected, a control biopsy was collected as described above; if the mare had positive cytology and/or growth of pathogens from the uterine swab, they were treated with intrauterine lavage and antimicrobials according to sensitivity testing before collecting the control biopsy. All mares were confirmed free from infection and inflammation at the time for collecting the control biopsy.

### Preparation of *E. coli *inocula

*E. coli *241 kept at -80°C was streaked on a blood agar plate (5% horse blood) and incubated for 24 h at 37°C. One colony was transferred to 2 ml of sterile Brain Heart Infusion broth (Fischer Scientific, Pittsburgh, PA, USA) and incubated overnight at 37°C. The overnight broth was serial diluted using sterile PBS to a concentration of 10^6 ^colony forming units (CFU) per ml and then diluted in 9 ml of sterile PBS to a final concentration of 10^6 ^CFU per 10 ml inoculum. The inocula were kept on ice until use (maximum 2 hours).

### Bacterial examination and exfoliative cytology of endometrial biopsies and swabs

Immediately after sampling, endometrial biopsies were divided in two pieces with a sterile scalpel. One part of the biopsy was dissected into small pieces (1-2 mm) and stored in RNAlater (Ambion, Austin, TX, USA) at 5°C for 24 h, followed by storage at -20°C until further processing. The other part of the biopsy and the uterine swab were streaked on blood agars (5% horse blood) and incubated aerobically for 24 h at 37°C. Bacterial growth was identified according to colony morphology, Hancock stain-morphology, haemolysis and catalase and potassium hydroxide (3% KOH) tests. Colonies were counted and scored: no growth/sterile: < 5 CFU, mild growth: 5-10 CFU, moderate growth: 11-50 CFU and heavy growth: > 50 CFU. Culture results were recorded as *E. coli*, beta-haemolytic *streptococci*, other uterine pathogens or no growth. When more than 3 different isolates were present, the culture was recorded as contamination. The biopsies and swabs were smeared on glass slides, which were dried at room temperature and stained with Diff-Quick ^® ^(Fisher Scientific), and evaluated by light microscopy (×400 magnification). Cytological classification of the uterine biopsies and swabs were based on numbers of PMNs present per 200 endometrial cells examined [23]. PMNs were counted and scored: no inflammation: 0-1 PMN, mild endometrial neutrophilia: 2 PMNs, moderate endometrial neutrophilia: 3-4 PMNs, and severe endometrial neutrophilia: > 5 PMNs.

### Blood sampling

Blood samples were obtained at 0, 3, 6, 12, 24, 48, 72, 96, 120, 168 h after inoculation and in the estrous following *E. coli *inoculation. Blood was drawn from the jugular vein using a Vacutainer^® ^system into tubes containing sodium citrate for the determination of plasma fibrinogen, tubes containing EDTA for analysis of white blood cell count (BD Vacutainer; BD-Vacutainer Systems, Plymouth, USA), tubes containing no additive (Butler Schein Animal Health) for SAA analysis, and PAXgene tubes (Qiagen, Valencia, CA, USA) for subsequent RNA isolation.

Analysis of the total white blood cell count (WBC) was performed using VetAutoread™ Hematology Analyzer (IDEXX, Westbrook, ME, USA) immediately after collection. Serum and plasma were prepared by centrifugation at 3500 × g at 4°C and stored at -20°C until analysis. Fibrinogen was determined by the Clauss method in an automated coagulometric analyzer (ACL 9000; Instrumentation Laboratory, Barcelona, Spain), and the concentration of SAA was determined by an automated analyzer (ADVIA 1650 Chemistry System; Bayer A/S, Lyngby, Denmark) using a commercially available immunoturbidometric assay (LZ test SAA; Eiken Chemical Co., Ltd., Tokyo, Japan) as described by Jacobsen et al. [24].

### qRT-PCR analysis

Total RNA was isolated from 60 mg of endometrial tissue stored in RNAlater using 650 μl TRIzol^® ^Reagent (Invitrogen, Carlsbad, CA, USA) as described by the manufacturer. SV Total RNA Isolation System (Promega, Mannheim, Germany) including DNAse treatment was used for clean-up of the extracted RNA. Total cellular RNA from leukocytes was isolated from approximately 2.5 ml whole blood collected into PAXgene tubes. The tubes were incubated at room temperature for 24 h and then stored at -20°C until assayed. Once thawed, total RNA was extracted and DNAse treated with the PAXgene blood RNA extraction kit (Qiagen) using manufacturer's protocol. RNA was quantified via spectrophotometry using a NanoDrop ND-1000 (Agilent Technologies, Palo Alto, CA, USA). All samples had 260/280 ratio of 1.95 or higher and 260/230 ratio of 2.0 or higher, and were used for further analysis. RNA samples (1000 ng/reaction for endometrial samples and 250 ng/reaction for blood samples) were reverse transcribed using a RT-PCR kit (Promega), Oligo dT (Promega) and random primers (R&D systems, Minneapolis, MN, USA). The total volume of each reaction was 25 μl. Reactions were incubated at 25°C for 10 min, heated at 42°C for 60 min, heated at 95°C for 5 min, then cooled to 4°C and stored at -20°C until qRT-PCR analysis.

The mRNA expression of *IL-1β*, *IL-1ra*, *IL-6*, *IL-8*, *IL-10*, *TNF-α *in leukocytes and endometrial tissue and the expression of *SAA *in endometrial tissue were measured by qRT-PCR. A specific primer for IL-1ra was designed using Primer3 http://frodo.wi.mit.edu/ (Table 1).

**Table 1:** Oligonucleotide primer sequences for amplification of various equine cytokines, *SAA *and reference genes

Target (gene)		Primer sequence (5'-3')	Product size (bp)	Source/Accesionnumber
*SAA*	F	CCT GGG CTG CTA AAG TCA TC	169	[25]

	R	AGG CCA TGA GGT CTG AAG TG		

*TNFa*	F	GGC CCA GAC ACT CAG ATC AT	73	[9]

	R	TTG GGG GTT TGC TAC AAC AT		

*IL1b3i*	F	CAG TCT TCA GTG CTC AGG TTT CTG	84	[9]

	R	CAT TGC CGC TGC AGT AAG T		

*IL-10i*	F	GCT GGA GGA CTT TAA GGG TTA C	76	[9]

	R	CAT CAC CTC CTC CAG GTA AAA		

*IL-8i*	F	CTT TCT GCA GCT CTG TGT GAA G	189	[9]

	R	GCA GAC CTC AGC TCC GTT GAC		

*β-actin*	F	CGT GGG CCG CCC TAG GCA CCA	243	AF035774.1

	R	TTG GCC TTA GGG TTC AGG GGG G		

*β-glucoronidase*	F	GCT CAT CTG GAA CTT TGC TGA TTT T	85	XM001493514.2

	R	CTG ACG AGT GAA GAT CCC CTT TT		

*GapdH*	F	GGG TGG AGC CAA AAG GGT CAT CAT	418	[26]

	R	AGC TTT CTC CAG GCG GCA GGT CAG		

*IL 6*	F	GGA TGC TTC CAA TCT GGG TTC AAT	65	[27]

	R	TCC GAA AGA CCA GTG GTG ATT TT		

*IL-1ra*	F	ACA AAT GTG GCT CCT CCA AG	88	NM_001082525

	R	TTT CAG AGC GTC AGA AGT GC		

All primers were commercially synthesized (Invitrogen). qRT-PCR was completed using SYBR Green PCR Master Mix (Applied Biosystems, Foster City, CA, USA) with the following cycling conditions: 95°C for 10 min; 45 cycles of 95°C for 10 s, 60°C for 10 s, 72°C for 30 s; 55-95°C for dissociation. The qRT-PCR reactions were performed in 96-well plates (for cDNA synthesized from leukocyte extracted RNA) and 384-well plates (for endometrial samples), with a final volume of 20 μl per reaction. Each reaction contained a diluted (10×) cDNA sample (4 μl), 10 μl SYBR green, 2 μl of each primer (forward and reverse, 10 μM) and 2 μl ddH_2_O. A no-template control (RNase-free water) was included for every qRT-PCR run. Samples were done in duplicates. Efficiency of amplification for each primer was monitored through the analysis of serial dilutions (10-fold). The melting curves of the amplified PCR products were obtained for confirmation of specific amplification. Negative controls containing no template (H_2_O) and non-reverse transcribed RNA were included to verify amplification of a single product. The product sizes of specific products were verified on a 1% agarose gel. A pool of all endometrial samples (from here on referred to as calibrator) was generated and added as internal control during each qRT-PCR analysis.

All gene amplifications from endometrial samples were normalized to *β-actin*, selected as the most stable reference gene across all uterine samples from a panel of three potential reference genes (glyceraldehydes-3-phosphate dehydrogenase (*GAPDH*), beta glucoronidase (*B-GUS*), beta-actin (*β-actin*)) analyzed using GeNorm [28]. *GAPDH *was used as an endogenous control gene for the leukocyte samples [29].

### Data analyses and statistical methods

Cycle threshold (Ct) values were obtained through the auto Ct function. Following efficiency correction, the mean threshold cycle (C_T_) was calculated and then normalized to the reference gene using delta (Δ) C_T_. The calibrator was used to carry out an additional normalization step in order to account for differences in amplification dynamics between PCR reactions between different PCR reaction plates. Changes in relative expression were calculated using the 2^-ΔΔCt ^method [30]. The specific transcripts are presented as n-fold change relative to pre-inoculation level (leukocytes) and estrous baseline levels (controlbiopsy, endometrium).

Outliers were defined as relative gene expression levels differing more than 3 × standard deviation, and were excluded for further data analyses. A high degree of individual variation in endometrial gene expression was observed, and in total 47 out of 752 (6%) gene expression levels were defined as outliers and excluded from data analyses.

The effect of intrauterine infusion of *E. coli *on repeated measurements of blood variables, SAA and cytokine mRNA expression in endometrial biopsies and leukocytes and cytological response was statistically analyzed using a repeated measures analysis of variance procedure in SAS (PROC MIXED). A first order autoregressive covariance structure was defined to take into account significant autocorrelation between measurements within mares. Differences in least squares mean estimates from the repeated measurement analyses were used to identify time points where the analyzed marker increased/decreased significantly from the pre-inoculation level. Bonferroni's multiple comparison procedure was used in order to control for Type I errors. To test if intrauterine infusion of PBS/*E. coli *elicited any upregulated endometrial gene expression of SAA and cytokines compared to estrous baseline levels, data was analyzed using the repeated measures analysis of variance as described above.

A non-parametric *t*-test (Mann Whitney *U *test) was used to identify specific time points where the gene expression differed significantly between resistant and susceptible mares and between different treatments (PBS and *E. coli*).

The effects of intrauterine infusion of PBS or *E. coli *on the presence on intrauterine fluid, bacterial growth of *E. coli, S. zooepidemicus *and other pathogens were statistically analyzed using linear logistic regression (PROC GENMOD) in SAS. A logit transformation of data was used to describe the relationship between the outcome and the explanatory variable. A generalized score test (Wald's test) was used in the type 3 analysis, and significant differences between the time points for sample collection were identified by using least square means. Goodness-of-fit tests were performed to control the model of analyses of a dichotomous outcome.

All values are presented as means ± standard error of the means (SEM). Assumptions were checked on residual plots and tested for normality. Initial inspection of the data revealed that SAA and cytokine mRNA expression varied markedly between individuals. Because variances were heterogenous, 2^-ΔΔCt ^values were log transformed, and geometric least square means statistically compared. All statistical calculations were made with the software SAS 9.2 (SAS Institute, Cary, NC, USA). Graphs were made using the software GraphPad Prism 5.0 (GraphPad Software Inc., La Jolla, CA, USA). The level of significance was set to p ≤ 0.05.

## Results

### Clinical and gynecological examination

Rectal temperature, heart rate, and respiratory frequency remained within normal limits for the entire study period. All mares were free from intrauterine fluid at the time of inoculation with PBS and *E. coli*. Inoculation of *E. coli *induced intrauterine fluid accumulation in a significantly higher number of mares than after inoculation with PBS (p = 0.001). None of the resistant mares had intrauterine fluid at any time point following PBS or *E. coli *inoculations, while two of the susceptible mares developed intrauterine fluid accumulation (at 3-72 h) after PBS inoculation and all seven susceptible mares had intrauterine fluid after *E. coli *infusion (four of the mares retained fluid at the last biopsy collection at 72 h).

### Microbiology

Eight out of 12 mares were *E. coli *culture positive with a few colonies from the uterine samples collected after *E. coli *infusion (Table 2). A high number of susceptible mares were culture positive for *S. zooepidemicus *following PBS infusion (five mares) or *E. coli *inoculation (four mares). Overall, significantly more susceptible than resistant mares were culture positive for *S. zooepidemicus *(p = 0.008), whereas no significant difference in the number of susceptible mares that were culture positive for *S. zooepidemicus *following PBS or *E. coli *inoculation was observed.

**Table 2:** Bacterial growth from uterine swabs and biopsies before (0 h) and 3, 12, 24 and 72 h after intrauterine infusion of PBS and *E. coli*

Mare ID	Class	0 h PBS	3 h PBS	12 h PBS	24 h PBS	72 h PBS	0 h Coli	3 h Coli	12 h Coli	24 h Coli	72 h Coli
A	R^a^	ng	ng	ng	ng	ng	ng	ng	ng	ng	+coli

B	R	ng	ng	ng	ng	ng	ng	++coli	ng	+coli	ng

C	R	ng	ng	ng	ng	ng	ng	ng	ng	ng	ng

D	R	ng	ng	ng	ng	ng	ng	++coli	ng	ng	+coli

E	R	ng	ng	ng	ng	ng	ng	ng	ng	+++Sez	+++Sez

F	S^b^	ng	ng	ng	ng	ng	ng	ng	+coli	+coli	+coli

G	S	ng	ng	ng	+++Sez	+++Sez	ng	ng	ng	+coli	ng

H	S	ng	ng	+++Sez^d^	+++Sez	+++Sez	ng	+coli	++coli/++Sez	+++Sez	+++Sez

I	S	ng	ng	ng	ng	+++Sez	ng	ng	+++Sez	+++Sez	+++Sez

J	S	ng	ng	+Sez	+++Sez	++Sez	ng	+Sez	+Sez	++coli/++Sez	++Sez

K	S	ng	ng	++OP^e^	++OP	ng	ng	ng	ng	ng	ng

L	S	ng	ng	ng	+++Sez	+++Sez	ng	+coli	+coli/++Sez	+++Sez	+++Sez

^a^resistant, ^b^susceptible, ^c^no growth, ^d^*S. zooepidemicus*, ^e^other pathogen, + mild growth, ++ moderate growth, **+++ **heavy growth

### Exfoliative cytology (data not shown)

All mares had moderate to severe neutrophilia of the endometrium immediately (3 h) and severe neutrophilia 12 h after *E. coli *inoculation. Resistant mares showed a significant decrease in neutrophilia at 72 h compared to previous samples (p < 0.001), whereas susceptible mares had a moderate to severe endometrial neutrophilia throughout the study period.

### Endometrial gene expression

At estrous baseline level (control biopsy) no difference in gene expression levels of any cytokines or *SAA *was observed between the resistant and susceptible mares.

A summary of all gene expression levels appear in the table in Additional file 1: Table S1.

### PBS inoculation

No significant changes in gene expression levels of any of the cytokines and SAA analysed was observed after PBS infusion when compared to estrous base line levels.

Compared to resistant mares, susceptible mares showed significant higher expression of the anti-inflammatory cytokine *IL-1ra *24 h (4 fold, p = 0.020) and lower gene expression of *IL-10 *(3 fold, p = 0.02) post inoculation with PBS.

### *E. coli *inoculation

Initial (3 h) significant upregulated gene expressions of *IL-1β *(p < 0.001), IL-6 (p < 0.001), *IL-8 *(p < 0.05) and TNF-α (p = 0.05) were observed in resistant mares, and *IL-6 *(p < 0.001) and *IL-1ra *(p < 0.001) in susceptible mares, when compared to estrous baseline levels.

### Endometrial gene expression response to *E. coli *vs. PBS

Inoculation with *E. coli *compared to inoculation with PBS elicited a significant upregulated endometrial gene expression (expressed relative to estrous baseline levels) pattern of *IL-1β *(p = 0.009), *IL-6 *(0.002) and *IL-8 *(p = 0.001) in both groups of mares. In resistant mares, the largest part of the variance in the data analysis was caused by the time-flow, whereas the gene expression levels among the susceptible mares depended strongly on the treatment (PBS or *E. coli*).

Initially (3 h) after infusion, resistant mares showed a significant upregulated gene expression of the pro-inflammatory cytokines *IL-1β *(12 fold, p = 0.015), *IL-6 *(41-fold, p = 0.036) and *IL-8 *(66-fold, p = 0.008) after *E. coli *infusion compared to PBS. The ratio *IL-1β *to its natural antagoinist *IL-1ra *(*IL-1β:IL-1ra*) was significantly increased at 3 h compared to PBS inoculation (53-fold, p = 0.008).

Susceptible mares had a significant increased gene expression of several pro-inflammatory cytokines after *E. coli *inoculation compared to the response after PBS inoculation; *IL-1β *at 3 h (5-fold, p = 0.03) and 72 h (11-fold, p = 0.013), *IL-6 *at 3 h (3-fold, p = 0.013), *IL-8 *at 72 h (30-fold, p = 0.002) and an increased expression of *IL-1ra *(14 fold, p = 0.03) 3 h post inoculation.

### Endometrial gene expression in susceptible mares vs. resistant mares

**Figure 2:**
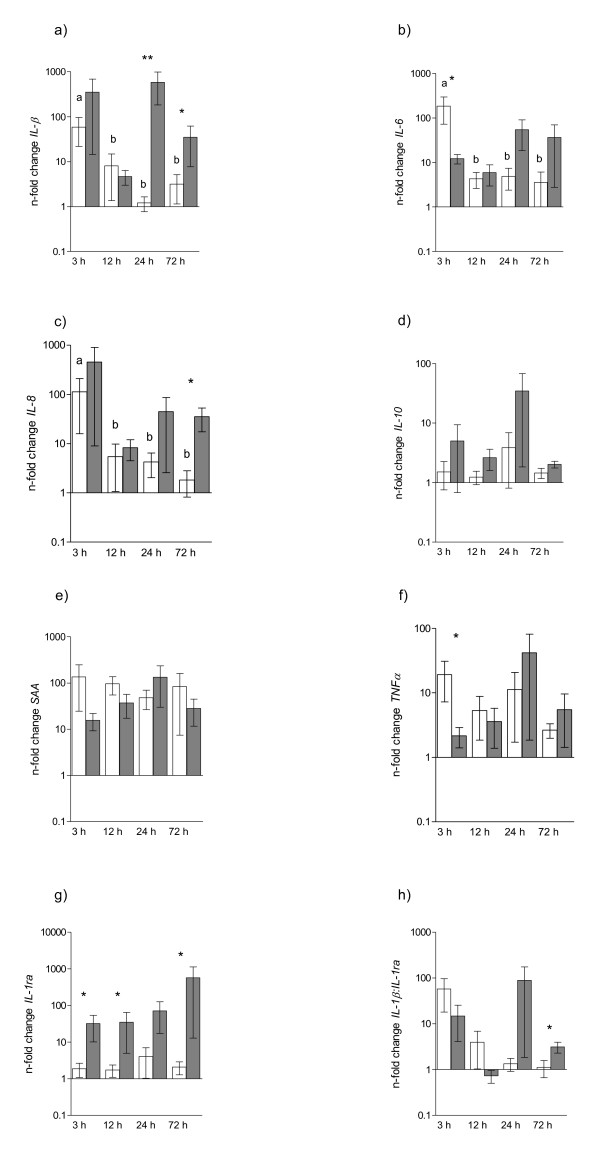
**mRNA transcripts of endometrial a) *IL-1β*, b) *IL-6*, c) *IL-8*, d) *TNF-α*, e) *SAA*, f) *IL-10*, g) *IL-1ra *and h) *IL-1β:IL-1ra *in mares after intrauterine infusion of *E. coli***. The gene expressions are normalized to *β-actin and displayed as n-fold change to *estrous baseline levels (mean ± sem). Asteriks indicate significant differences between resistant (white bars) and susceptible (grey bars) mares. *p < 0.05. Different letters above the bars indicate significant differences between the time points within the group of mares (p < 0.05).

Susceptible mares showed increased levels of *IL-1β *24 h (10-fold, p = 0.020), 72 h (10-fold, p = 0.02), and *IL-8 *at 72 h (13-fold, p = 0.0024) (Figure 2a, c) after *E. coli *infusion compared to resistant mares. The expression of *IL-1ra *was significantly increased in susceptible versus resistant mares at all time points (range: 6-20 fold, p < 0.05) (Figure 2g). Susceptible mares showed immediately (3 h) after inoculation a significantly lower expression of *IL-6 *(15-fold, p = 0.02) and *TNF-α *(9-fold, p = 0.015), but no difference at 12, 24 or 72 h (Figure 2b, d). Susceptible mares had a decreased ratio of *IL1β:IL-1ra *immediately after *E. coli *inoculation (3 h) (4-fold, p = 0.05), but an increased ratio was observed at the end of the study period (72 h) (4-fold, p = 0.05) compared to resistant mares (Figure 2h). No significant difference in endometrial mRNA transcripts for *SAA *between the two groups was observed (Figure 2e).

Using Spearman correlation analysis, the anti-inflammatory *IL-10 *was significantly positive correlated to mRNA transcripts of *IL-1ra*, with the highest correlation observed in resistant mares (r = 0.99) (susceptible mares: r = 0.7).

### Endometrial gene expression related to uterine infection

Endometrial biopsies with growth of *S. zoopidemicus *24 and 72 h after PBS and *E. coli *infusion had significantly higher endometrial gene expression of *IL-1β *(8-fold) and *IL-1ra *(14-fold) compared to biopsies with no growth, or positive for mild growth of *E. coli *and other pathogens (p < 0.05). The degree of inflammation evaluated by exfoliative cytology 72 h after *E. coli *infusion in susceptible mares correlated to the upregulated *IL-8 *expression in these mares at the same timepoint (p = 0.05).

### Systemic APR and gene expression of cytokines in circulating leukocytes (data not shown)

The plasma concentration of SAA, fibrinogen and WBC showed no significant difference after *E. coli *inoculation. No significant up- or down-regulated gene expression of *IL-1β*, *IL-6*, *IL-8*, *IL-10 *and *IL-1ra *could be detected at the different time points for blood collection after *E. coli *inoculation compared to pre-inoculation levels. The expression of *TNF-*α was significantly upregulated in resistant as well as susceptible mares at 168 h compared to 120 h (2-fold, p = 0.05). Susceptible mares showed a lower gene expression of *IL-10 *compared to resistant mares at day 7 (2-fold, p = 0.02), and an upregulated expression of *IL-1ra *48 h after inoculation (4-fold, p = 0.02).

## Discussion

The present investigation demonstrates varying levels of gene expression by equine endometrial cells as assessed by an array of inflammatory mediators produced in response to intrauterine infusion of *E. coli *and PBS independent of the status of mares being resistant or susceptible.

### Endometrial gene expression of cytokines and SAA

To our knowledge this is the first study to demonstrate that repeated endometrial biopsy collection aiming at evaluating the endometrial inflammatory response can be performed within the same estrous cycle without causing significant trauma to the endometrium affecting the results. Comparing the gene expression analyses after PBS infusions with the mares own control at estrous levels (control biopsy) showed that repeated endometrial biopsy collection (within 72 h) had no effect on clinical signs or endometrial gene expression of any of the cytokines or *SAA *analyzed. Our findings are in contrast to a study by Palm et al. where an evaluation of the endometrial inflammatory response in reproductively normal pony mares 12 h after intrauterine infusion of PBS, seminal plasma and semen extender, revealed a significant increased gene expression of several pro-inflammatory cytokines (*IL-1β*, *IL-6 *and *TNF-α*) [31]. The conflicting findings may be due to differences in protocols and handling of data. Data in the present study was normalized to the reference gene *β-actin *and an internal control ("calibrator") as described by Livak & Schmittgen [30]. The calibrator was included in every qRT-PCR run to standardize the analysis and minimize variations among the different qRT-PCR plates. In the study by Palm et al., the gene expression was only normalized to reference genes, which may explain the contrary findings.

Both groups of mares exhibited a pro-inflammatory response to *E. coli *infusion measured as a fast increase in endometrial gene expression of interleukins (*IL-1β, IL-6, IL-8*) initially after infusion (3 h). The production of IL-1β in tissues is thought to contribute to local effects such as fibrosis, tissue matrix breakdown and the influx of inflammatory cells to initiate the inflammatory cascade [32]. Endotoxin and other microbial products are strong stimuli for IL-1 transcription [32], which may explain the increased *IL-1β *gene expression level in mares immediately after *E. coli *inoculation (3 h) and in mares culture positive for *S. zooepidemicus*. Only a few mares were culture positive for a very mild load of *E. coli *late in the study period, which might not have been sufficient to induce *IL-1β *expression.

The control of pro-inflammatory responses to avoid excessive immune activation by bacteria including the effects of *IL-1β*, depends on anti-inflammatory mediators as IL-10 [33] and IL-1ra [34]. In the present study, no significant up- or down-regulation of *IL-10 *was observed, even though a dampened gene expression of cytokines (*IL-1β*, *IL-6, TNF-α *and *IL-8*), normally regulated by IL-10, was observed primarily in resistant mares. This is in contrast to our earlier experiment using a higher dose of *E. coli*, which resulted in a significantly increased gene expression of *IL-10 *initially after infusion [9]. The IL-10 response is thought to reflect the strength of the preceeding inflammatory response [35], and the constant low expression of *IL-10 *might be explained by the relative low bacterial challenge observed in the present study.

Resistant mares had a higher expression of *IL-6 *and TNFα initially after *E. coli *infusion compared to susceptible mares as part of the pro-inflammatory cascade. One of their main functions is to initiate and regulate the synthesis of acute phase proteins (APP) crucial for the APR to infection [36,37]. An early up-regulation has been suggested as a first line of defense to avoid uterine bacterial invasion [38], even though correlation between the low gene expression in susceptible mares and bacterial growth from uterine samples could not be established in the present study.

In the resistant mares the levels of pro-inflammatory cytokines peaked initially (3 h) after inoculation (*IL-1β, IL-6, IL-8 *and *TNF-α*), whereas susceptible mares showed a prolonged and sustained inflammatory response (*IL-1β, IL-8, IL-1ra*). Previous studies by Fumuso and co-workers showed that mares susceptible to persistent endometritis had significant upregulated cellular immune response (*IL-1β, IL-6, TNF-α *and *IL-8*) and down-regulated anti-inflammatory activity (*IL-10*) compared to resistant mares at estrous baseline levels [11,12]. In the present study no differences in endometrial gene expression of cytokines and *SAA *in the estrous subsequent to *E. coli *infusion were found between the two groups of mares; all mares were culture-negative for uterine pathogens and inflammation at estrous baseline levels, which could explain the identical baseline levels among the groups.

Mares classified as susceptible to persistent endometritis had a prolonged inflammatory response and showed significant higher gene expression levels of IL-1β, *IL-8, IL-1ra *and IL-1β:IL-1ra ratio 72 h after *E. coli *infusion compared to resistant mares. Expression of *IL-1β *and *IL-1ra *was significantly elevated in mares with heavy growth of *S. zooepidemicus *compared to mares with no bacterial growth or mild growth of *E. coli *from uterine samples. These findings correlate well with observations from cows where a significant upregulated expression of *IL-1β *and the competitor to the receptor, *IL-1ra*, has been observed in cows with post partum uterine disease [39,40].

The upregulated endometrial gene expression of *IL-1β, IL-8 *and *IL-1ra *72 h after bacterial challenge could be a part of the impaired and delayed uterine clearance scenario associated with reduced myometrial activity observed in susceptible mares. Fluid, pathogens and inflammatory products will likely accumulate within the uterus lumen [6,17,41] and may result in a sustained inflammatory response. The response is driven by an upregulated expression of IL-8 responsible for the continuing migration of PMN's into the uterine lumen [42], and IL-1β and IL-1ra responsible for initiating and down-regulating the pro-inflammatory response, respectively. The upregulated *IL-8 *expression observed in susceptible mares at 72 h correlated with their severity of endometrial inflammation (exfoliative cytology) at this time point. IL-8 is a potent chemoattractant [43], and PMNs play a crucial role in clearing uterine inflammation by stimulating the synthesis and release of PGF-2α from the endometrium activating uterine contractility [44], and by stimulating phagocytosis and bactericidal activity [45]. Uterine PMNs from susceptible mares have been demonstrated to be fully functional if given an optimal environment. Impaired opsonization from uterine secretions may cause dysfunctional PMNs with impaired phagocytosis in susceptible mares [46]. The delayed uterine clearance causing accumulation of intra uterine fluid is thought to play a key role in the sustained inflammatory response and may have a negative effect on opsonization [6]. An unbalanced relationship between expression of IL-1β and its antagonist IL-1ra could be an indicator for an imbalance between pro-inflammatory and modulating cytokines, which has been demonstrated to be of pathogenic importance in chronic inflammatory diseases [47].

### Microbiology

No pathogenic microorganisms could be isolated from endometrial swabs and biopsies at the time of infusion with PBS or *E. coli*, respectively. The numbers of *E. coli *recovered from uterine samples from resistant and susceptible mares after *E. coli *inoculation were low to moderate. Three mares (two resistant and one susceptible) were culture positive for only very few colonies of *E. coli *at the 72 h collection (< 10 CFU), which may be regarded as insignificant [48] or at least as a very mild infection. Two mares had initially after *E. coli *infusion no growth from uterine swabs and biopsies, but had mild growth of *E. coli *(5-10 CFU) from the uterine samples collected 72 h after inoculation. Nielsen demonstrated a higher sensitivity when using an endometrial biopsy compared to a uterine swab sample for bacterial cultures [23]. A low number of bacteria within the uterine lumen of some of the mares in the present study may however have been too low to be obtained on the uterine swab sample and biopsy, and can explain why some mares were culture negative immediately after inoculation, and became culture positive late in the study period (72 h). The rapid clearance of the inoculated *E. coli *is in accordance with a study by Nicolakopoulos and Watson in which resistant mares cleared *S. zooepidemicus *within the first 48 hours post inoculation [49].

The relative low inoculum dose of *E. coli *and the current stage of the reproductive cycle may explain the low number of *E. coli *recovered subsequently compared to a previous study by our group [9]. In that study mares were inoculated with a high dose of *E. coli *(10^9 ^CFU) in diestrous, where the endometrium was under influence of progesterone and thus may have had reduced immune responses [16]. Increased mobilization of PMNs in uteri of estrous animals likely leads to the elimination of bacteria before infection becomes established [50].

Six out of the seven susceptible mares developed uterine infections after PBS inoculation; five mares had heavy growth of *S. zooepidemicus *12*-*24 h after inoculation, and one mare was infected with another pathogen (*Panthoea agglomerans*). All mares remained infected throughout the study period (12-72 h). None of the uterine samples collected from resistant mares after PBS infusion contained any pathogens. Mares culture positive for *S. zooepidemicus *and other pathogens after PBS infusion were treated with antimicrobials according to sensitivity testing and intrauterine flushings, and were confirmed culture-negative and cytology negative prior to *E. coli *infusion. Surprisingly, four of the five susceptible mares initially positive for *S. zooepidemicus *after PBS infusion again became culture-positive for *S. zooepidemicus *when infused with *E. coli*, as did one resistant mare, which was culture-negative after PBS infusion. This could indicate that *S. zooepidemicus *is able of causing persistent endometrial infections, a notion which is supported by work that demonstrated that *S. zooepidemicus *in chronically infected mares are localized deep within the endometrium [51]. The present observations suggest that *S. zooepidemicus *is capable of establishing a persistent infection within the endometrial tissue, and it is possible to "activate" the growth of *S. zooepidemicus *by infusing an inflammatory-inducing substance (PBS or *E. coli*. This is supported in a recent study, which demonstrates that inactive/dormant *S. zooepidemicus *in persistently infected mares can be activated (Petersen et al. 2011, unpublished observations).

### Cytology and intrauterine fluid accumulation

Measurement of intrauterine fluid accumulations using ultrasonography and PMN counts from uterine swabs or uterine fluid smears represent common methods to evaluate inflammation of the equine endometrium [52]. PMN migration into the uterus peaks about 6 h after experimental introduction of bacteria to the uterine lumen, and normally the response will remain elevated for at least 72 h [4,5,53]. In the present study both groups of mares had a high cytological score immediately after the bacterial inoculation (3 to 24 h), whereas this was significantly lower in the resistant mares at 72 h post inoculation. Mares with intrauterine fluid had a tendency to show a more severe cytological response compared to mares without intrauterine fluid.

The cause of presence, distribution and retention of uterine fluid is multifactorial and includes multiparity and age, poor perineal conformation and lymphatic drainage [54]. In the present study the susceptible mares were older (mean age 19 years) than the resistant mares (mean age 6 years), and it is possible that age-related poor perineal conformation and impaired lymphatic drainage had an impact on development of susceptibility towards persistent inflammation. Alghamdi et al. demonstrated that susceptible mares had a significant higher NO concentration in uterine secretions and higher endometrial gene expression of nitric oxide synthases (iNOS) 13 h after insemination compared to resistant mares [55]. In cows uterine concentration of nitric oxide has also been associated with uterine inflammation and infectious endometritis [56]. NO is an inflammatory mediator, and function as the main mediator of smooth muscle relaxation in different organs including the uterus [57], and the intrauterine fluid accumulation in the susceptible mares in the present study may be due to impaired myometrial contractility partly caused by high concentrations of nitric oxide.

The etiology of susceptibility to persistent endometritis is multifactorial. Several age- and parity related factors predispose the aged mare to susceptibility, and repeated uterine infections may also contribute to the degree of susceptibility. Histological endometrial lesions correlate to resistance/susceptibility to persistent endometritis, with increased age and Kenney biopsy score highly associated with accumulation of inflammatory cells and fluid in response to an inflammatory stimulatory agent [58] (Woodward et al., manuscript submitted 2011). A recent study demonstrated that mares can changes their susceptibility status based on fluid retention and cytology from one breeding season to the next. The endometrial quality in these mares did not change. These finding suggest that the duration of endometrial inflammation and fluid accumulation in response to inflammation induction is dynamic, and that mares can improve their resistance against persistent inflammation (Woodward et al., manuscript submitted 2011). Nevertheless, an optimal experimental design would have matched resistant and susceptible mares with regards to age. Under the condition of this study, this was not possible, dueto the strong correlation between age and susceptibility to PBIE. Some of the observed changes in cytokine gene expression may therefore, have been caused by advanced age in the susceptible group.

### Systemic APR and gene expression of cytokines in circulating leukocytes

Lipopolysaccharides and infections with Gram-negative bacteria are known to be potent inducers of APRs and inflammation [59,60]. Systemic responses have previously been demonstrated in mares with experimentally induced endometritis using a high dose of *E. coli *[9], and in bitches with pyometra [61]. In cattle initiation of a systemic APR is a dominant feature of post partum endometritis [62,63]. The APR has previously been found to depend both on the lipopolysaccharide dose and on individual variation [64], and to correlate well with the degree of uterine contaminants in cows [62] and ewes with clinical endometritis [65]. No significant increase or decrease beyond the reference limits in blood parameters analyzed was observed in the present study, which is in contrast to observations in mares inoculated with a high dose of *E. coli *[9]. These contrary findings suggest that intra uterine inoculation of a moderate dose of *E. coli *in resistant and susceptible mares does not initiate a systemic APR. A significant higher gene expression of *TNF-α *in leukocytes was observed in both groups (2-fold) at 168 h compared to 120 h, may be a consequence of the cycle stage of the mares. Progesterone may have an inhibitory effect on circulating leucocytes' gene expression of *TNF-α *as observed in uterine derived leucocytes [66]. The significantly increased anti-inflammatory response in susceptible mares (*IL-1ra*) 48 h after *E. coli *infusion compared to resistant mares may reflect the increased endometrial anti-inflammatory response observed in the susceptible mares.

None of the mares developed clinical signs of endotoxemia, which is in contrast to a previous study where mares became endotoxemic within the first 3-12 h after diestrous intrauterine inoculation with a high dose (10^9 ^CFU) of the identical *E. coli *strain (241) as used in the present study [9]. Infectious endometritis or persistent post breeding endometritis do not elicit a systemic physiologic response [67] therefore; the dose used in the present study is likely to be more representative for *E. coli *induced endometritis in a broodmare.

## Conclusions

In conclusion, the results of the current investigation demonstrated that inflammatory cytokines are expressed in the equine endometrium in a time-related manner during an experimentally induced infectious endometritis with a peak initially after bacterial infusion. The systemic APR to intrauterine inoculation of *E. coli *appears dose-dependent due to the undetected response following a low-dose inoculation in the present study.

Endometrial mRNA transcripts of pro-inflammatory cytokines as a response to endometritis are finely regulated, with an initial high expression level followed by normalization within a short period of time (12-24 h) in resistant mares. Susceptible mares had a prolonged expression of pro-inflammatory cytokines and what appears to be an unbalanced relationship between pro- and anti-inflammatory cytokines (*IL-1β:IL-1ra*), supporting the hypothesis that an unbalanced endometrial gene expression of inflammatory cytokines might play an important role in the pathogenesis of persistent endometritis. Furthermore, it is hypothesized that persistent infections with *S. zooepidemicus *influence the gene expression of pro- and anti-inflammatory cytokines suggesting that a persistent *S. zooepidemicus *infection may play a central role in the pathogenesis of persistent endometritis. Further investigations are required to determine the exact role of persistent infections in susceptible mares.

## Competing interests

The authors declare that they have no competing interests.

## Authors' contributions

MC participated in the design and coordination of the study, carried out the study, performed the statistical analyses, and wrote the manuscript. EW and MRP helped carrying out clinical parts of the study. SJ, HLJ, AMB and MHT conceived the study and participated in its design and coordination. All authors read and approved the final manuscript.

## Supplementary Material

Additional file 1 **Gene expression profiles in resistant (R) and susceptible (S) mares after PBS and *E. coli *inoculation expressed relative to estrous base line levels**. ^a^Not significant, ^b^increase. Click here for file
