# Supplementary material for: Inflammatory responses to induced infectious endometritis in mares resistant or susceptible to persistent endometritis
Source: BMC Vet Res. 2012 Mar 29;8:41. doi: 10.1186/1746-6148-8-41 (PMC3368729; doi:10.1186/1746-6148-8-41)
Supplement: Additional file 1 — Gene expression profiles in resistant (R) and susceptible (S) mares after PBS and E. coli inoculation expressed relative to estrous base line levels. aNot significant, bincrease. [file 1746-6148-8-41-S1.DOC]

| Gene | Treat | Time | Group | Means fold change | S.E.M | P values | Change in expression | |
| --- | --- | --- | --- | --- | --- | --- | --- | --- |
| *IL1β* | PBS | 3 | R | 8.5 | 0.2 | NS^a^ | NS |  |
| *IL1β* | PBS | 12 | R | 1.5 | 0.3 | NS | NS |  |
| *IL1β* | PBS | 24 | R | 1.0 | 0.1 | NS | NS |  |
| *IL1β* | PBS | 72 | R | 1.0 | 0.1 | NS | NS |  |
| *IL1β* | PBS | 3 | S | 3.5 | 0.1 | NS | NS |  |
| *IL1β* | PBS | 12 | S | 2.9 | 0.3 | NS | NS |  |
| *IL1β* | PBS | 24 | S | 1.25 | 0.1 | NS | NS |  |
| *IL1β* | PBS | 72 | S | 1.4 | 0.1 | NS | NS |  |
|  |  |  |  |  |  |  |  |  |
| *IL1β* | *E. coli* | 3 | R | 28.0 | 10.1 | <0.01 | ↑^b^ |  |
| *IL1β* | *E. coli* | 12 | R | 2.8 | 0.8 | NS | NS |  |
| *IL1β* | *E. coli* | 24 | R | 1.5 | 0.1 | NS | NS |  |
| *IL1β* | *E. coli* | 72 | R | 1.6 | 0.6 | NS | NS |  |
| *IL1β* | *E. coli* | 3 | S | 82.0 | 12.8 | NS | NS |  |
| *IL1β* | *E. coli* | 12 | S | 5.5 | 0.1 | NS | NS |  |
| *IL1β* | *E. coli* | 24 | S | 65.1 | 17.2 | <0.05 | ↑ |  |
| *IL1β* | *E. coli* | 72 | S | 51.07 | 16.1 | <0.01 | ↑ |  |
|  |  |  |  |  |  |  |  |  |
| *IL6* | PBS | 3 | R | 13.0 | 7.3 | NS | NS |  |
| *IL6* | PBS | 12 | R | 2.7 | 2.5 | NS | NS |  |
| *IL6* | PBS | 24 | R | 2.0 | 0.8 | NS | NS |  |
| *IL6* | PBS | 72 | R | 0.4 | 0.3 | NS | NS |  |
| *IL6* | PBS | 3 | S | 4.6 | 1.4 | NS | NS |  |
| *IL6* | PBS | 12 | S | 1.4 | 0.5 | NS | NS |  |
| *IL6* | PBS | 24 | S | 2.5 | 1.9 | NS | NS |  |
| *IL6* | PBS | 72 | S | 0.8 | 0.2 | NS | NS |  |
|  |  |  |  |  |  |  |  |  |
| *IL6* | *E. coli* | 3 | R | 59.3 | 12.4 | <0.01 | ↑ |  |
| *IL6* | *E. coli* | 12 | R | 1.6 | 1.9 | NS | NS |  |
| *IL6* | *E. coli* | 24 | R | 2.6 | 1.2 | NS | NS |  |
| *IL6* | *E. coli* | 72 | R | 2.5 | 0.21 | NS | NS |  |
| *IL6* | *E. coli* | 3 | S | 13.0 | 2.4 | <0.01 | ↑ |  |
| *IL6* | *E. coli* | 12 | S | 4.2 | 1.3 | NS | NS |  |
| *IL6* | *E. coli* | 24 | S | 77.5 | 25.4 | NS | NS |  |
| *IL6* | *E. coli* | 72 | S | 26.4 | 8.9 | NS | NS |  |
|  |  |  |  |  |  |  |  |  |
| *IL8* | PBS | 3 | R | 4.6 | 2.7 | NS | NS |  |
| *IL8* | PBS | 12 | R | 9.8 | 0.8 | NS | NS |  |
| *IL8* | PBS | 24 | R | 0.5 | 0.8 | NS | NS |  |
| *IL8* | PBS | 72 | R | 0.2 | 0.1 | NS | NS |  |
| *IL8* | PBS | 3 | S | 1.9 | 0.9 | NS | NS |  |
| *IL8* | PBS | 12 | S | 5.4 | 1.4 | NS | NS |  |
| *IL8* | PBS | 24 | S | 0.7 | 0.1 | NS | NS |  |
| *IL8* | PBS | 72 | S | 1.1 | 0.5 | NS | NS |  |
|  |  |  |  |  |  |  |  |  |
| *IL8* | *E. coli* | 3 | R | 23.4 | 5.4 | <0.01 | ↑ |  |
| *IL8* | *E. coli* | 12 | R | 3.9 | 1.2 | NS | NS |  |
| *IL8* | *E. coli* | 24 | R | 2.0 | 1.1 | NS | NS |  |
| *IL8* | *E. coli* | 72 | R | 2.0 | 1.9 | NS | NS |  |
| *IL8* | *E. coli* | 3 | S | 6.1 | 4.8 | NS | NS |  |
| *IL8* | *E. coli* | 12 | S | 4.0 | 1.7 | NS | NS |  |
| *IL8* | *E. coli* | 24 | S | 48.8 | 15.8 | NS | NS |  |
| *IL8* | *E. coli* | 72 | S | 26.3 | 9.4 | <0.05 | ↑ |  |
|  |  |  |  |  |  |  |  |  |
| *IL10* | PBS | 3 | R | 2.8 | 0.8 | NS | NS |  |
| *IL10* | PBS | 12 | R | 1.6 | 0.3 | NS | NS |  |
| *IL10* | PBS | 24 | R | 2.1 | 0.8 | NS | NS |  |
| *IL10* | PBS | 72 | R | 3.5 | 1.4 | NS | NS |  |
| *IL10* | PBS | 3 | S | 0.5 | 0.2 | NS | NS |  |
| *IL10* | PBS | 12 | S | 1.6 | 0.5 | NS | NS |  |
| *IL10* | PBS | 24 | S | 1.0 | 0.3 | NS | NS |  |
| *IL10* | PBS | 72 | S | 1.9 | 0.7 | NS | NS |  |
|  |  |  |  |  |  |  |  |  |
| *IL10* | *E. coli* | 3 | R | 1.6 | 0.5 | NS | NS |  |
| *IL10* | *E. coli* | 12 | R | 1.2 | 0.7 | NS | NS |  |
| *IL10* | *E. coli* | 24 | R | 1.4 | 0.7 | NS | NS |  |
| *IL10* | *E. coli* | 72 | R | 8.0 | 5.2 | NS | NS |  |
| *IL10* | *E. coli* | 3 | S | 2.0 | 0.3 | NS | NS |  |
| *IL10* | *E. coli* | 12 | S | 2.1 | 0.7 | NS | NS |  |
| *IL10* | *E. coli* | 24 | S | 22.14 | 6.72 | NS | NS |  |
| *IL10* | *E. coli* | 72 | S | 1.8 | 0.6 | NS | NS |  |
|  |  |  |  |  |  |  |  |  |
| *IL1ra* | PBS | 3 | R | 3.5 | 0.5 | NS | NS |  |
| *IL1ra* | PBS | 12 | R | 1.7 | 0.5 | NS | NS |  |
| *IL1ra* | PBS | 24 | R | 70.7 | 18.7 | NS | NS |  |
| *IL1ra* | PBS | 72 | R | 17.1 | 10.7 | NS | NS |  |
| *IL1ra* | PBS | 3 | S | 2.7 | 0.8 | NS | NS |  |
| *IL1ra* | PBS | 12 | S | 4.1 | 2.8 | NS | NS |  |
| *IL1ra* | PBS | 24 | S | 3.8 | 1.2 | NS | NS |  |
| *IL1ra* | PBS | 72 | S | 3.0 | 1.2 | NS | NS |  |
|  |  |  |  |  |  |  |  |  |
| *IL1ra* | *E. coli* | 3 | R | 1.7 | 0.6 | NS | NS |  |
| *IL1ra* | *E. coli* | 12 | R | 3.9 | 1.2 | NS | NS |  |
| *IL1ra* | *E. coli* | 24 | R | 1.4 | 0.9 | NS | NS |  |
| *IL1ra* | *E. coli* | 72 | R | 1.6 | 0.3 | NS | NS |  |
| *IL1ra* | *E. coli* | 3 | S | 18.5 | 3.7 | <0.01 | ↑ |  |
| *IL1ra* | *E. coli* | 12 | S | 16.8 | 8.8 | NS | NS |  |
| *IL1ra* | *E. coli* | 24 | S | 30.4 | 8.2 | <0.01 | ↑ |  |
| *IL1ra* | *E. coli* | 72 | S | 58.7 | 8.78 | NS | NS |  |
|  |  |  |  |  |  |  |  |  |
| *TNFa* | PBS | 3 | R | 3.91 | 1.1 | NS | NS |  |
| *TNFa* | PBS | 12 | R | 1.7 | 0.7 | NS | NS |  |
| *TNFa* | PBS | 24 | R | 1.1 | 0.3 | NS | NS |  |
| *TNFa* | PBS | 72 | R | 1.8 | 0.4 | NS | NS |  |
| *TNFa* | PBS | 3 | S | 1.36 | 0.3 | NS | NS |  |
| *TNFa* | PBS | 12 | S | 0.8 | 0.3 | NS | NS |  |
| *TNFa* | PBS | 24 | S | 1.3 | 0.2 | NS | NS |  |
| *TNFa* | PBS | 72 | S | 0.9 | 0.4 | NS | NS |  |
|  |  |  |  |  |  |  |  |  |
| *TNFa* | *E. coli* | 3 | R | 6.3 | 0.17 | <0.01 | ↑ |  |
| *TNFa* | *E. coli* | 12 | R | 1.6 | 0. | NS | NS |  |
| *TNFa* | *E. coli* | 24 | R | 1.9 | 0.8 | NS | NS |  |
| *TNFa* | *E. coli* | 72 | R | 5.75 | 0.9 | <0.05 | ↑ |  |
| *TNFa* | *E. coli* | 3 | S | 8.6 | 6.7 | NS | NS |  |
| *TNFa* | *E. coli* | 12 | S | 32.7 | 11.7 | NS | NS |  |
| *TNFa* | *E. coli* | 24 | S | 43.1 | 21.8 | NS | NS |  |
| *TNFa* | *E. coli* | 72 | S | 5.0 | 2.8 | NS | NS |  |
|  |  |  |  |  |  |  |  |  |
| *SAA* | PBS | 3 | R | 8.31 | 3.2 | NS | NS |  |
| *SAA* | PBS | 12 | R | 3.3 | 0.7 | NS | NS |  |
| *SAA* | PBS | 24 | R | 1.83 | 0.8 | NS | NS |  |
| *SAA* | PBS | 72 | R | 0.63 | 0.1 | NS | NS |  |
| *SAA* | PBS | 3 | S | 2.8 | 1.2 | NS | NS |  |
| *SAA* | PBS | 12 | S | 12.1 | 3.1 | NS | NS |  |
| *SAA* | PBS | 24 | S | 34.4 | 6.5 | NS | NS |  |
| *SAA* | PBS | 72 | S | 28.1 | 11.1 | NS | NS |  |
|  |  |  |  |  |  |  |  |  |
| *SAA* | *E. coli* | 3 | R | 4.2 | 1.4 | NS | NS |  |
| *SAA* | *E. coli* | 12 | R | 12.1 | 3.81 | <0.05 | ↑ |  |
| *SAA* | *E. coli* | 24 | R | 6.3 | 3.4 | NS | NS |  |
| *SAA* | *E. coli* | 72 | R | 7.3 | 2.49 | NS | NS |  |
| *SAA* | *E. coli* | 3 | S | 8.6 | 0.6 | <0.01 | ↑ |  |
| *SAA* | *E. coli* | 12 | S | 48.4 | 8.4 | NS | NS |  |
| *SAA* | *E. coli* | 24 | S | 69.1 | 9.2 | <0.001 | ↑ |  |
| *SAA* | *E. coli* | 72 | S | 54 | 11.4 | <0.05 | ↑ |  |
